# The Benefits and Costs of Using Social Distancing to Flatten the Curve for COVID-19

**DOI:** 10.1017/bca.2020.12

**Published:** 2020-04-28

**Authors:** Linda Thunström, Stephen C. Newbold, David Finnoff, Madison Ashworth, Jason F. Shogren

**Affiliations:** 1 Department of Economics, University of Wyoming, 1000 E. University Avenue, Laramie, WY 82071, USA; 2 Department of Economics, University of Wyoming, 1000 E. University Avenue, Laramie, WY 82071, USA; 3 Department of Economics, University of Wyoming, 1000 E. University Avenue, Laramie, WY 82071, USA; 4 Department of Economics, University of Wyoming, 1000 E. University Avenue, Laramie, WY 82071, USA, e-mail: lthunstr@uwyo.edu (L. T.), snewbold@uwyo.edu (S. C. N.)

**Keywords:** benefits, coronavirus, costs, COVID-19, flatten the curve, social distancing., D12, D18, D61, D78, D81, E17, E61, F13, H12, I15, I18, O11

## Abstract

We examine the net benefits of social distancing to slow the spread of COVID-19 in USA. Social distancing saves lives but imposes large costs on society due to reduced economic activity. We use epidemiological and economic forecasting to perform a rapid benefit–cost analysis of controlling the COVID-19 outbreak. Assuming that social distancing measures can substantially reduce contacts among individuals, we find net benefits of about $5.2 trillion in our benchmark case. We examine the magnitude of the critical parameters that might imply negative net benefits, including the value of statistical life and the discount rate. A key unknown factor is the speed of economic recovery with and without social distancing measures in place. A series of robustness checks also highlight the key role of the value of mortality risk reductions and discounting in the analysis and point to a need for effective economic stimulus when the outbreak has passed.

## 1 Introduction

Are the attempts to slow down the rate of COVID-19 infections by social distancing worth the cost? Because no proven treatment or vaccine exists for COVID-19, the only effective measure available to control the virus and protect public health is to reduce the frequency of close contacts among people. Governments around the world have issued unprecedented policies and guidelines to increase social distance within and across countries. The goal is to save lives by reducing the pace and extent of COVID-19 infections (“flatten the curve”), and to avoid overtaxing nations’ health care infrastructure as symptomatic people seek medical care. In USA, the federal government has issued guidelines urging citizens to avoid gatherings of 10 or more people to help reduce community spread. Schools, universities, and daycare centers have temporarily ceased operations, playgrounds and other public spaces have been sealed off, cultural events have been canceled, tourist attractions including Broadway and Disney World have closed, and national sports leagues have suspended or canceled their seasons. Furthermore, the federal government has imposed travel restrictions on Canada, China, Iran, Mexico, and a wide range of European countries to reduce external exposure to the virus.

While these social distancing measures save lives, they also impose significant costs on society. The resulting contraction of economic activity puts vulnerable low-income workers in jeopardy, and recent forecasts point to historic declines in economic output in the coming months, despite large fiscal and monetary stimulus. On March 31, Goldman Sachs presented an economic forecast for 2020 in which they predict U.S. Gross Domestic Product (GDP) will shrink by 6.2 % this year, largely due to the combined effects of the mortality, morbidity, associated productivity impacts of the epidemic, and the social distancing measures being adopted to control it (Goldman Sachs, 2020a). These economic impacts, along with the public health benefits, should be considered at the national level when evaluating any rational risk reduction policy. As the direct economic costs of social distancing become increasingly salient to households and businesses, decision-makers and the general public can benefit from systematic policy evaluations to help determine whether those costs are justified by the value of the lives saved. If the public health benefits are not effectively communicated, voluntary compliance with social distancing guidelines may decline faster than otherwise, which could undermine the effectiveness of such policies (Maharaj & Kleczkowski, 2012).

We compare the benefits and costs in USA of flattening the curve through social distancing policies. Using a standard epidemiological model, we measure benefits by the number of lives saved from reducing the spread of COVID-19 through social distancing. The difference in mortality without and with social distancing provides our projections of the number of lives saved, for which we calculate economic benefits using current estimates of the average willingness to pay for reductions in mortality risk. We measure costs by the difference in present value of GDP lost in a scenario without social distancing to GDP lost with social distancing. The loss of GDP under both scenarios is defined by an immediate decline in GDP and subsequent recovery over time. Our benefit cost analysis is the outcome of the comparison of the present value of lives saved (benefits) to the present value of the difference in GDP lost without and with social distancing (costs).

Based on this comparison, we find that social distancing policies likely do not constitute an overreaction to COVID-19. In a variety of plausible scenarios based on the best available information as of 3 April 2020, we find that the economic benefits of lives saved outweigh the value of the projected losses of GDP by about $5.2 trillion using a 3 % discount rate and a 30 year planning horizon. To test the robustness of the main result, we examine the sensitivity of the estimated net benefits to a wide range of alternative model assumptions. We estimate the break-even values (i.e., those that produce net benefits equal to zero) for the main model parameters including relative GDP recovery rates without and with social distancing, the discount rate, COVID-19 infection rates, human mortality rates, contact rates, the immediate decline in GDP, and the medical capacity threshold. A key finding in the sensitivity analysis is the joint influence of the assumed discount rate and planning horizon. We find that if one were to extend the planning horizon beyond our benchmark of 30 years, the present value of lost GDP – which never declines to zero according to the economic forecasts without and with social distancing – would eventually overcome the value of lives saved, all of which occur in the first year of the policy forecast. In this case, a higher discount rate gives a larger net present value, all else equal, because all the benefits occur immediately. This is in contrast to applications of benefit–cost analysis to climate change policies, in which many observers have argued for low discount rates and long planning horizons to give due weight to the far future benefits of policies with high present day costs (Giglio *et al*., 2015).

We now turn to our methods. While numerous parameters drive the analysis, the three key building blocks to focus on are: (i) how infectious COVID-19 can be and how this translates into mortality rates without and with social distancing, which determines the number of lives saved under the policy; (ii) the immediate decline in GDP and the rate of recovery without and with social distancing, which determines the cost of the policy, and (iii) the presumed discount rate and planning horizon, which matters because under social distancing most lives will be saved now whereas the GDP loss will be extended into the future.

## 2 Methods

We begin by examining the benefit side of the analysis. Benefits from social distancing are measured in terms of total value of lives saved. Measuring the benefits from social distancing requires that we capture the extent to which social distancing succeeds in reducing the contact rate between individuals, and the degree to which that is sufficient to prevent the health care system from becoming overwhelmed, since an overwhelmed health care system implies higher mortality rates due to limited access to adequate care. To estimate the infectiousness of COVID-19 (i.e., the average number of secondary infections the first infected person causes) and how this translates into mortality rates without and with social distancing, we model the spread of COVID-19 using a standard SIR (Susceptible Infectious Recovered) framework used in epidemiology. An SIR model tracks the numbers of susceptible, infected, and recovered individuals over the course of an infectious disease outbreak (Kermack & McKendrick, 1927; Hethcote, 2000).

The equations of motion for the number of susceptible, infected, and recovered individuals in the population are as follows:(1)
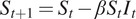

(2)
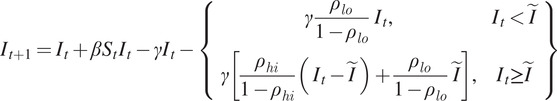

(3)
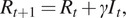
where 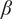 reflects the rate of contacts among individuals in the population and the likelihood that an encounter between an infected individual and a susceptible individual will result in spreading the virus to the susceptible individual, and 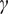 is the recovery rate of infected individuals. The components of Equations (1)–(3) excluding the term in braces comprise the standard SIR model. The term in braces accounts for the fact that some infected individuals die, and are removed from the compartment of infected individuals (Keeling & Rohani, 2008, p. 34). We model the effect of the number of infected individuals exceeding the capacity of the health care system with a differential mortality rate for excess cases: 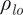 is the probability that an infected individual dies before recovering when the health care system threshold is not exceeded (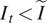), and 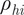 is the probability of death for the excess infected cases when the threshold is exceeded (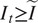). Accounting for mortality, the basic reproduction number in this model is 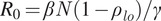, which is the relationship we use to calibrate the contact rate 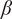. Note that 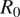
captures the contagiousness of the disease; it pertains to early in the outbreak when negligible fraction of population has been infected.

In our benchmark specification, we assume a basic reproduction number (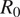) of 2.4, which is a central value among estimates from several epidemiological studies based on early rates of spread in China and elsewhere (Ferguson *et al*., 2020; Liu *et al*., 2020). An 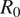 of 2.4 means that each infected person is expected to spread the virus to 2.4 others, on average. We use an average infectious period (which is the reciprocal of the recovery rate, 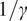) of 6.5 days, which is consistent with reported cases of COVID-19 from early January to early February 2020 (Lauer *et al*., 2020; Liu *et al*., 2020). These assumptions roughly match those used by the Centers for Disease Control and Prevention (CDC), based on reporting of their modeling results in popular media. We set the initial number of infections to 4165, which was the CDC official estimate of infected individuals in USA on 17 March 2020. Based on estimates of the influence of similar social distancing measures taken in Australia to combat the spread of the 1918 Spanish flu, and assuming these measures are adopted widely and maintained for the duration of the outbreak, we assume social distancing will reduce the average contact rate among individuals by 38 % (Caley *et al*., 2008). The value of reduced mortality risk (Value of Statistical Life, VSL) is taken to be $10 million, which is consistent with U.S. federal agency guidelines^1^ and recent syntheses of the mortality risk valuation literature (see Viscusi, 2018; Kniesner & Viscusi, 2019). As Viscusi (2018, p. 25) notes “[t]here is no single VSL.” The $10 million value reflects the average money-risk preferences of exposed populations over different economic conditions. For a more detailed discussion on the challenges of fine-tuning the selection of the VSL, see Chapter 8 in Viscusi (2018).

To account for the possibility of overwhelming the U.S. health care system, we make a critical assumption that the system has sufficient resources to provide adequate treatment for one half of the maximum number of individuals who would be infected at any one time in an uncontrolled scenario, with no social distancing to slow the spread of the virus. In the benchmark case, this threshold is 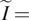 36 million infected people. We assume that the mortality rate for infected individuals who are treated when the threshold is not exceeded (i.e., when the health care system is not overwhelmed) is 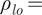 0.5 %, and the mortality rate above the threshold is 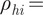 1.5 %. While these assumptions were made independently, the first assumption appears to be in line with initial findings by the Harvard Global Health Institute (HGHI, 2020) and reporting in the New York Times on U.S. hospital bed capacity in the face of COVID-19 (Sanger-Katz *et al*., 2020), and the assumed mortality rates appear to lie within the range employed by the CDC modeling, as reported in popular media.

**Figure 1:**
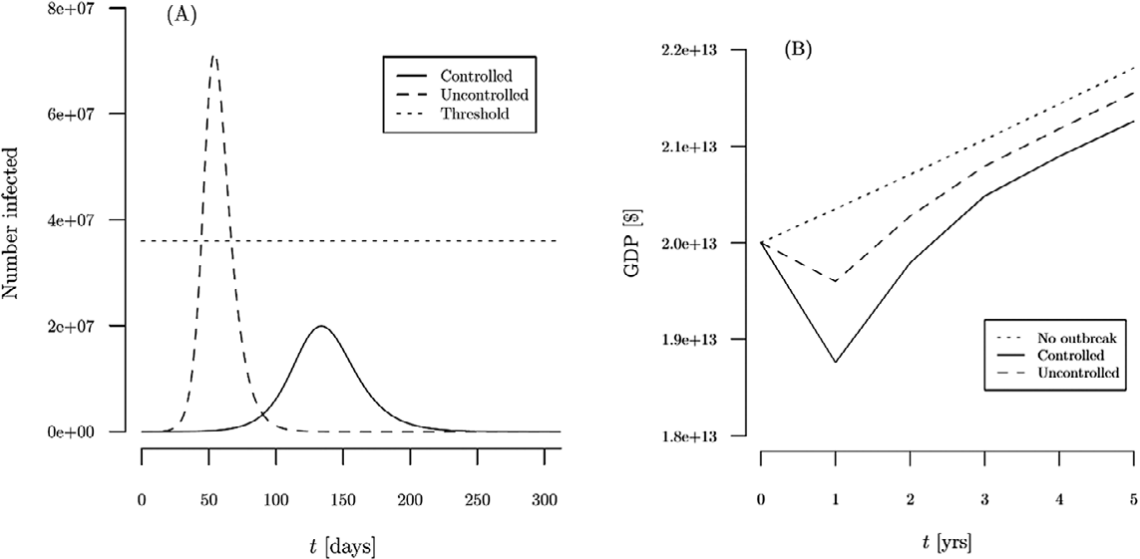
Projections of coronavirus infections (*a*) and GDP losses (*b*) for benchmark uncontrolled and controlled outbreak scenarios.

Now consider the cost side. We measure costs to social distancing as lost GDP. The economic consequences of the pandemic are felt in an immediate decline of GDP and subsequent increasing but lower than counterfactual (without-pandemic) GDP as the economy recovers over time and converges to the baseline projected growth rate (Figure 1). The key to the cost estimates is the assumptions about the magnitude of the shock to GDP and the recovery rate of the economy without and with social distancing. Note that even in the absence of social distancing, the economy is likely to enter a recession, due to an overwhelmed health care system and loss of productivity due to absenteeism and severe health consequences from a widespread virus. In the absence of the pandemic, we assume GDP would have grown at a constant rate of 1.75 % per year for the foreseeable future (“baseline projected”), following Goldman Sachs’ estimate of potential GDP growth in USA, which also in line with the estimate by the United States Congressional Budget Office (CBO, 2020).

For the controlled scenario (with social distancing), we adopt the most recent economic forecast by Goldman Sachs, which incorporates the anticipated economic effects of the outbreak itself and from the social distancing measures currently being adopted in response to it (Goldman Sachs, 2020a). We assume that GDP will immediately decline by 6.2 % in 2020, and then grow 5.5, 3.5, and 2.0 % in the following three years and 1.75 % per year thereafter. This is broadly similar to the recovery time observed after the Spanish flu in USA (OECD, 2003).

Recent estimates of the immediate economic impact from the COVID-19 pandemic when social distancing is not implemented – due to the large number of excess deaths, loss of productivity due to sick days, and the inordinate strain on the health care system during the span of the outbreak – range from an immediate decline in GDP of 1.5–8.4 % (McKibbin & Fernando, 2020). We assume outcomes at the higher end of this range are relatively less likely, so for the uncontrolled scenario (without social distancing) we choose an immediate decline of 2.0 % for our benchmark case (as we show below, less optimistic assumptions would strengthen our main result). We are not aware of a forecast of GDP growth in subsequent years if the epidemic were left uncontrolled, so we assume the same proportional rate of growth after the immediate decline in GDP in the uncontrolled scenario as forecasted by Goldman Sachs for the controlled scenario. We assume the ratio of the gap between the baseline (no outbreak of COVID-19) GDP trend and the uncontrolled GDP path and the gap between the baseline trend and the controlled GDP path in all subsequent years is equal to the initial ratio of the GDP trend gaps. Whether recovery will be slower or faster with social distancing than without depends largely on the extent to which the spread of the virus can be slowed and how effective any additional economic stimulus will be at boosting the economy in the short and medium term after the outbreak has passed.

We write the net present value of social distancing as the value of the lives saved by social distancing minus the present value of GDP lost due to social distancing:(4)
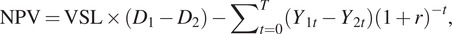


where 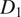 and 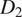 are the number of deaths without and with social distancing, 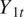 and 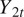 are the forecasted levels of GDP in year 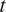 without and with social distancing, 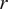 is the discount rate, and 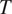 is the planning horizon.

## 3 Results

The projections of infections over time in our benchmark uncontrolled (without social distancing) and controlled (with social distancing) scenarios are shown in panel (*a*) of Figure 1, and the associated projections of GDP are shown in panel (*b*). Panel (*a*) indicates that social distancing measures sufficient to decrease the average contact rate among individuals by 38 % can reduce the peak of the infection curve by more than half. This would avoid overwhelming the health care system and keep the average mortality rate at the lower level of 0.5 %.

Consider first our main result – the net benefits of social distancing are positive. Table 1 presents the main BCA results for social distancing by way of comparison between the uncontrolled and controlled scenarios. Based on our SIR model, the total number of infections is projected to reach 287 million without social distancing and 188 million with social distancing. When combined with the differential mortality rates when the health system capacity threshold is exceeded versus when not, the difference between the infection curves translates into about 1.24 million lives saved. Using a $10 million value of reduced mortality risk (VSL) for the lives saved, the benefits of social distancing are $12.4 trillion. The cost of social distancing is the difference in present value terms of the GDP losses without ($6.49 trillion) and with ($13.7 trillion) the policy, which is $7.21 trillion. The main result is in the bottom row: under our benchmark assumptions, social distancing generates net benefits of about $5.16 trillion.

**Table 1:** Benchmark outcomes for the uncontrolled scenario (without social distancing) and controlled scenario (with social distancing).

	Uncontrolled	Controlled
Infections (million)	287	188
Deaths (million)	2.18	0.941
Present value of GDP loss (trillion US$)	6.49	13.7
Value of lives lost (trillion US$)	21.8	9.41
Net benefits (trillion US$)		5.16

Benchmark parameter values: 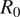
= 2.4, infectious period = 6.5 days, low mortality rate = 0.5 %, high mortality rate = 1.5 %, reduction in contact rate = 38 %, VSL = $10 million, uncontrolled initial GDP decline = 2.0 %, controlled initial GDP decline = 6.2 %, medical capacity threshold = 36 million infected, equal proportional rates of recovery in uncontrolled and controlled scenario, discount rate = 3.0 % year^−1^, planning horizon = 30 years.

At the time of this study, there is still uncertainty about the ultimate spread, severity, and duration of COVID-19 in USA, and about the severity and duration of the economic impacts from the pandemic, both if the virus were to be left uncontrolled and if social distancing measures are maintained. As an illustration of how rapidly predictions and knowledge are changing, between March 20 and March 31 Goldman Sachs revised downward their U.S. GDP growth forecast for 2020 from 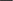
3.8 % to 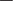
6.2 %.

This points to the importance of using sensitivity analysis to examine the robustness of our benchmark results to changes in key assumptions. First, we repeat the analysis assuming the speed of recovery in the uncontrolled scenario would be faster than the controlled scenario, and then assuming that the recovery would be slower. Next, we calculate break-even values for each of the key model parameters, holding all other parameters at their benchmark levels. We also construct break-even curves (i.e., zero-net benefit isoquants) for several pairs of key model parameters.

**Figure 2:**
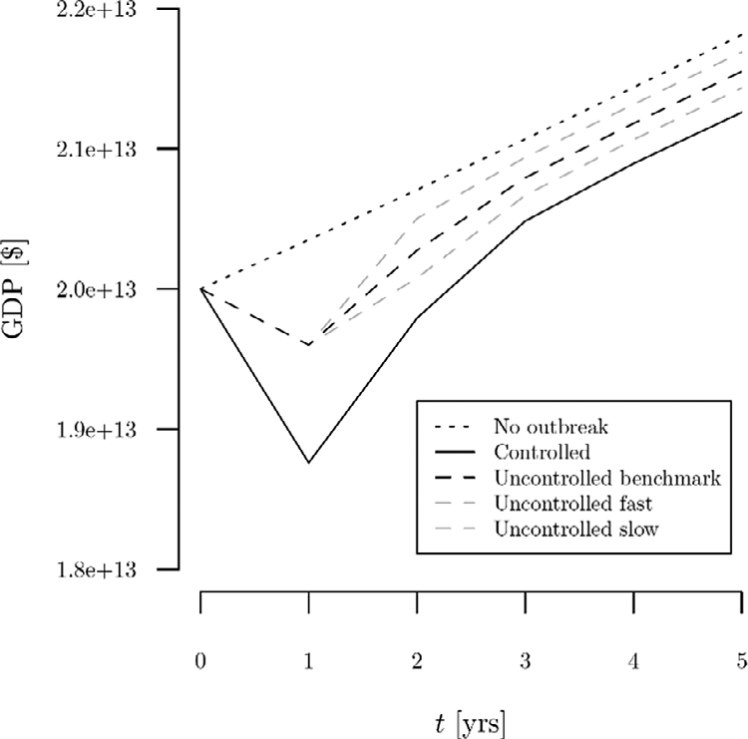
Alternative assumptions about the speed of recovery in the uncontrolled outbreak scenario.

Table 2 (top section) shows the results of our sensitivity analysis based on alternative assumptions about the speed of economic recovery in the uncontrolled (without social distancing) scenario. We consider a faster recovery case (second column) and a slower recovery case for the uncontrolled scenario (third column). These variations produce the GDP paths in Figure 2. Note that in our benchmark case the ratio of the uncontrolled to controlled outbreak GDP gaps relative to the baseline (no-outbreak) scenario in the initial year is (2.0 + 1.75)/(6.2 + 1.75). For the faster and slower recovery cases, we assume ratios equal to the square and the square root of the benchmark ratio. While arbitrary, we believe these variations to be plausible and chosen mainly to illustrate the influence of the relative speed of economic recovery between the two scenarios on the resulting net benefits. To assess the sensitivity of our results to the recovery rate of the economy, we keep the GDP path in the controlled (with social distancing) scenario and the immediate decline of GDP in the uncontrolled scenario the same as in the benchmark case; we only vary the subsequent GDP levels in the uncontrolled scenario. (We examine ceteris paribus changes in the initial GDP declines in our break-even analysis below.) The second and third columns in the top section of Table 2 show the key outcomes from these alternative cases: the net benefits of social distancing are $2.11 trillion and $7.78 trillion in the faster and slower recovery uncontrolled outbreak cases. These results highlight the importance of well-calibrated economic stimulus to avoid an unnecessarily prolonged recession: speeding up the recovery can save trillions of dollars.

**Table 2:** Central results and break-even values for key parameters under alternative economic recovery assumptions for uncontrolled (without social distancing) and controlled (with social distancing) scenarios.

	Equal rate of recovery	Faster uncontrolled recovery	Slower uncontrolled recovery
Central results			
Value of reduced mortality (trillion US$)	12.4	12.4	12.4
Present value of GDP loss (trillion US$)	7.27	10.3	4.64
Net benefits of control (trillion US$)	5.16	2.11	7.78
Break-even values			
Discount rate (% year^−1^)	None	1.7	None
Planning horizon (year)	63	38	None
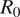	1.33, 4.46	1.58, 3.71	1.20, 5.53
Low mortality rate (%)	None	None	None
High mortality rate (%)	0.81	1.22	0.46
Reduction in contact rate (%)	19.6	28.0	13.4
VSL (million US$)	5.85	8.30	3.75
Uncontrolled initial GDP decline (%)	None	0.45	None
Controlled initial GDP decline (%)	8.5	7.0	11.0
Threshold (million infected)	54.6	42.5	None

Benchmark parameter values: basic reproduction number 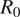 = 2.4, infectious period = 6.5 days, low mortality rate = 0.5 %, high mortality rate = 1.5 %, reduction in contact rate = 38 %, VSL = $10 million, uncontrolled initial GDP decline = 2.0 %, controlled initial GDP decline = 6.2 %, medical capacity threshold = 36 million infected, equal proportional rates of recovery in uncontrolled and controlled scenario, discount rate = 3.0 % year^−1^, planning horizon = 30 years. Faster uncontrolled recovery rate is twice as fast as controlled recovery rate. Slower uncontrolled recovery rate is half as fast as controlled recovery rate.

The bottom section of Table 2 shows the results of our break-even analyses. We calculated break-even values under all three assumptions about the relative speed of economic recovery, our benchmark case and the cases with faster and slower growth in the uncontrolled scenario. A key finding in the sensitivity analysis is dependency on the planning horizon and the discount rate. In our benchmark case, we discount future benefits and costs at a rate of 3 % per year. This is the lower of the two discount rates (the other being 7 %) recommended by the Office of Management and Budget for economic analyses conducted by U.S. federal government agencies.^2^ In our sensitivity analysis, we examine higher and lower discount rates encompassing 7 % and beyond.

We find that the net benefits remain positive for all (non-negative) discount rates when social distancing recovery is relatively fast or equal to uncontrolled GDP growth. If social distancing recovery is sufficiently slower than uncontrolled GDP growth, net benefits are negative if the discount rate is less than 1.7 %. Because the gap between the uncontrolled and controlled GDP paths never closes in our benchmark and faster uncontrolled scenario cases (a conservation assumption) if the planning horizon of the analysis is extended far enough into the future, the present value of lost GDP can overtake the value of lives saved in the current year. We see this if the rates of recovery are equal or if there is a faster uncontrolled recovery. In contrast to climate change policy analysis, in this setting benefits (lives saved) come early while costs extend into the future, so net benefits are lower when the discount rate is low and the planning horizon is long.

**Figure 3:**
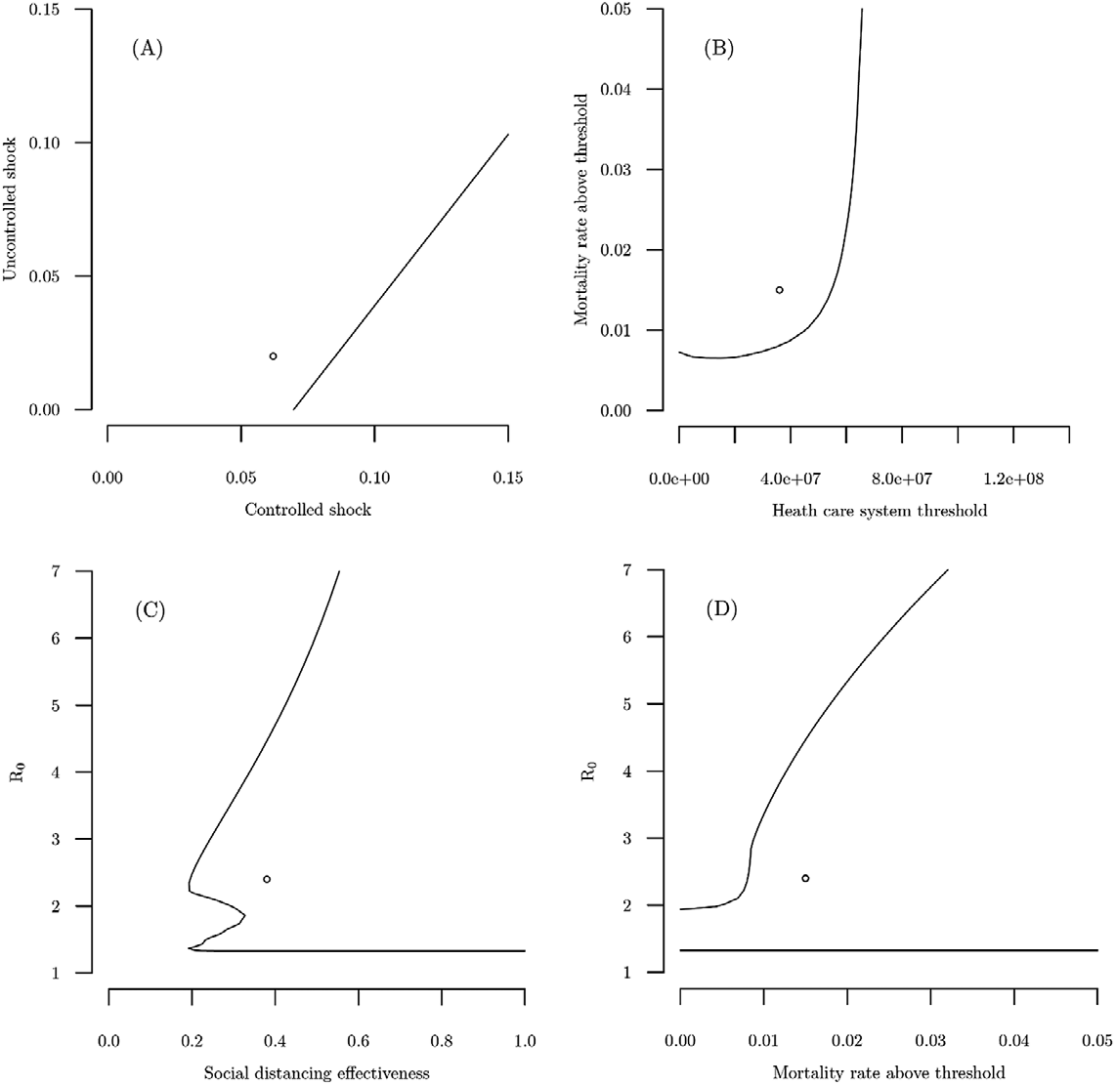
Break-even curves for four pairs of model parameters. Circle markers indicate the benchmark levels of the parameters labeled on the *x*- and *y*-axes, and lines trace out parameter combinations where net benefits equal zero.

We also extend the break-even analysis to allow key parameter values to pairwise vary. Figure 3 includes graphs that show break-even curves (zero net benefit isoquants) for four pairs of key model parameters. The circle marker indicates the benchmark levels for the parameters labeled on the *x*- and *y*-axes (those that produce our main results in Table 1), and the lines trace out the parameter combinations that produce net benefits equal to zero, holding all other model parameters fixed at their benchmark levels. Because net benefits are positive in our benchmark case, all combinations of the two parameters in the region on the same side of the lines as the circle marker also yield positive net benefits, and all combinations of the parameters on the other side of the lines yield negative net benefits.

Table 2 shows that in our benchmark case net benefits would be negative if the immediate decline in GDP with social distancing is 8.5 % or more, while any decline in GDP without social distancing (up to the benchmark value of the decline with social distancing) still yields positive net benefits. Panel (*a*) in Figure 3 demonstrates the sensitivity of the break-even value for ranges in both shocks. Net benefits from social distancing remain positive as long as the uncontrolled shock to GDP is more severe than the controlled shock, or if the magnitude of the controlled shock is larger than the uncontrolled shock by amounts to the left of the contour. For example, if the uncontrolled immediate decline in GDP were 5 %, net benefits of social distancing remain positive as long as the controlled immediate decline in GDP were less in absolute terms than roughly 10 %, all else equal. The general conclusion is that if the immediate decline in GDP from social distancing is significantly greater than that without social distancing – to the right of the breakeven contour – then net benefits become negative.

The sensitivity of the benchmark scenario to our assumptions about the medical capacity threshold, 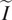, and mortality rate under an overwhelmed medical system, 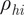, is indicated by the break-even values in Table 2 and illustrated by the curve in panel (*b*) of Figure 3. Holding all other parameters at their benchmark levels, net benefits would be negative if the medical capacity threshold is above 54.6 million, or the high infectious case mortality rate is below 0.81 %. The break-even curve shows that all high mortality rates larger than our benchmark level, and all medical capacity threshold levels smaller than our benchmark level produce positive net benefits, while sufficiently smaller high mortality rates or sufficiently larger medical capacity threshold levels would produce negative net social benefits. Note that net benefits in all three scenarios are always positive regardless of the (low) mortality rate before the medical system is overwhelmed, all else equal.

Because the death rate due to COVID-19 is substantially higher for elderly individuals (Wu & McGoogan, 2020), the break-even value for the average value of mortality risk, given by the VSL, is important. Some economic models suggest that individuals’ willingness-to-pay for mortality risk reductions declines with age (Shepard & Zeckhauser, 1984). Some empirical studies have estimated an inverted U-shape in which VSL rises and falls for older cohorts (Aldy & Viscusi, 2008), while other studies point to different conclusions based on efficiency (Evans & Smith, 2006; Kniesner *et al*., 2006) and fairness (see Viscusi, 2018, Chapter 5, p. 107). Given we use an average VSL of $10 million, we now consider the break-even VSL. If a substantially lower VSL is appropriate for older individuals, such that the average VSL were below $5.85 million, our benchmark result would change, as shown in Table 2. Another way to view the break-even VSL is that, in our benchmark case, the extensive social distancing measures currently underway amount to spending an average of $5.85 million per life saved. In a recent study, Greenstone and Nigam (2020) use age-varying values of reduced mortality risk, and estimate the benefits of social distancing for COVID-19 at $4.51 million per life saved, lower than our break-even VSL.

The sensitivity of net benefits to 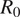 and social distancing effectiveness (assumed to reduce the average contact rate among individuals by 38 % at benchmark) is shown in Table 2 and panels (*c*) and (*d*) of Figure 2. Table 2 shows that as long as 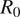 lies within the range 1.33 to 4.46, all else equal, net benefits from social distancing remain positive. Panel (*c*) extends this to show that a wide range of 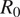 and social distancing effectiveness levels above our benchmark levels also produce positive net benefits.^3^ The break-even curve in panel (*d*) shows that net benefits remain positive for larger values of both 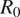 and the mortality rate with an overwhelmed health care system.

**Figure 4:**
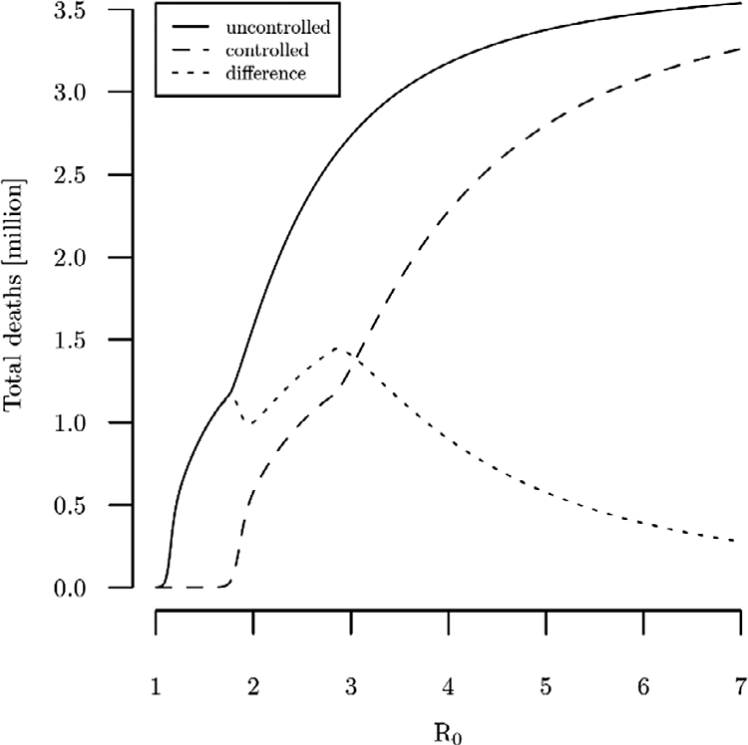
Total deaths in uncontrolled and controlled scenario varying *R*_0_ with all other parameters held fixed at their benchmark levels.

The curves in panels (*c*) and (*d*) also indicate that if 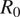 is sufficiently large then net benefits can be negative. That low 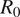 values can lead to negative net benefits is not surprising, since the number of deaths due to infection increases in 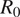, as shown in Figure 4. The reason that sufficiently high 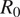 levels also can lead to negative net benefits is that if 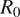 is large enough then a social distancing effectiveness at our benchmark level of 38 % is not sufficient to substantially reduce the number of individuals who get infected. As implied by Figure 4, the fraction of the population that becomes infected increases in 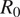 at a decreasing rate, so for high enough 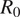 values a 38 % reduction from social distancing still leaves a large fraction of the population infected. This result matters given the early release article from the CDC (Sanche *et al*., 2020) that estimated that the median 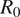 might be as high as 5.7, using new case reports from China. Figure 3, panel (*c*) shows that to ensure net benefits are positive at this larger level of 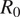, the effectiveness of social distancing needs to reduce the contact rate by around 50 %, all else equal.

Our key social distancing policy parameter is the reduction in the contact rate. For the benchmark recovery scenario, as long as that reduction is greater than 19.6 % (roughly one half of our assumed reduction), net benefits of social distancing remain positive. If the uncontrolled recovery is faster, the breakeven reduction rises to 28 %, while it falls to 13.4 % if the uncontrolled recovery is faster.

## 4 Discussion

We conduct a rapid benefit–cost analysis of social distancing measures to control the COVID-19 outbreak, based on the best available estimates of disease spread and impacts on the economy. We conclude that social distancing likely generates net social benefits. In our benchmark case, which we view as the most plausible case among those we examined, the present value of net benefits from social distancing amount to $5.16 trillion.

Because much uncertainty still surrounds both the characteristics of COVID-19 and the impact on the economy with and without social distancing, a key component of our study is the suite of sensitivity analyses that we conducted to examine the robustness of our benchmark results. The take-home message from our sensitivity analysis is that there are large regions in the parameter space that produce positive net social benefits, but there are also regions that do not. Some of our benchmark parameters are reasonably close to their break-even levels, which suggests that positive net benefits are not assured. This highlights the importance of rapid data collection, accurate policy evaluations, and responsive policy adjustments as our understanding of the public health and economic consequences of the pandemic improves. The value of information and policy flexibility is highest when the social net benefits of the available policy options are most ambiguous.

Furthermore, many of our model parameters are endogenous to public behavior and policies, such as compliance with social distancing, increases of the capacity of the health care system, and stimulus of the economy. This points to the importance of an active government in addressing the COVID-19 crisis. For instance, an important policy challenge will be to ensure that the large-scale coordination game underlying social distancing is successful in “flattening the curve” of the outbreak during a fairly limited time period – the economic forecast by Goldman Sachs that underlies our benchmark case implicitly assumes that economic activity will pick up again in the summer months of 2020. For that to materialize, people need to be willing and able to sustain effective social distancing measures, even in the face of a severe economic contraction.

Our analysis has several limitations. First, we assessed only a single policy package, which is intended to represent the full suite of self-quarantine and other social distancing measures currently being adopted in USA. Our finding of positive net benefits indicates that this response is better than the alternative of taking no measures to control the outbreak. While there may be other combinations of policies that could be adopted for this pandemic or in the future, we leave those for future work. Second, we focus exclusively on estimating the overall net benefits of social distancing, which means we ignore the likely distributional impacts of such measures. It stands to reason that the most vulnerable groups in society will be the hardest hit. For example, the labor-intensive service industry will be disproportionately affected by these policies, which will lead to mass layoffs of low-income workers. It also is likely that economically disadvantaged groups will suffer the most severe adverse health consequences from COVID-19. In principle, the asymmetric impact of the epidemic and economic burdens of the policy responses can be mitigated with appropriate redistributions of resources. A detailed analysis of the distribution of benefits and costs among income, age, rural versus urban regions, and other relevant individual or community characteristics could help to refine the control measures adopted in future pandemics. Third, we do not consider how current social distancing measures might affect the probability of a second wave of COVID-19 infections in the future by preventing the development of herd immunity (Matrajt & Longini Jr, 2012). Instead, we implicitly assume that aggressive social distancing measures buy enough time to develop and distribute cost-effective COVID-19 treatments or vaccines, should a second wave occur.

Finally, our paper has been purposefully focused. Future work should consider other potentially important consequences of the pandemic, including the associated decline in levels of pollution, a possible spike in domestic violence and suicides, and other non-market social impacts.

## Footnotes

1 See U.S. Department of Transportation, “Guidance on treatment of the economic value of statistical life (VSL) in U.S. Department of Transportation Analyses.” https://www.transportation.gov/regulations/guidance-treatment-economic-value-statistical-life-us-department-transportation-analyses, and U.S. Environmental Protection Agency (EPA), “Guidelines for preparing economic analyses.” https://www.epa.gov/sites/production/files/2017-08/documents/ee-0568-50.pdf

2 See Office of Management and Budget, (OMB), “Circular A-4.” https://www.whitehouse.gov/sites/whitehouse.gov/files/omb/circulars/A4/a-4.pdf.

3 Using a stylized network model designed to represent a small U.S. town, Glass et al. (2006) reported that “For influenza as infectious as 1957–58 Asian flu (≈50 % infected), closing schools and keeping children and teenagers at home reduced the attack rate by >90 %.”

## Supplementary material

For supplementary material accompanying this paper visit http://dx.doi.org/10.1017/bca.2020.12. click here to view supplementary material
