# Supplementary material for: The Benefits and Costs of Using Social Distancing to Flatten the Curve for COVID-19
Source: J Benefit Cost Anal. 2020 Apr 28:1–17. doi: 10.1017/bca.2020.12 (PMC7242774; doi:10.1017/bca.2020.12)
Supplement: Supplementary file 1 [file S2194588820000123sup001.pdf]

```r
#==============================================================================
# corona-bca.R
#==============================================================================

# NOTE: This script should be placed in a folder named 'code' and another folder
# named 'output' should be created at the same directory level.

#------------------------------------------------------------------------------
# PRELIMINARIES:
#------------------------------------------------------------------------------
if(1==1){
  # Clear console and environment:
  cat('\014')
  rm(list = setdiff(ls(), lsf.str()))

  # install.packages('tikzDevice')
  library(tikzDevice)

  # Streamlined functions for formatting:
  s2 <- function(x){return(sprintf('%-.2f',x))}
  s3 <- function(x){return(sprintf('%-.3f',x))}
  s4 <- function(x){return(sprintf('%-.4f',x))}

  # Define paths for file handling:
  this.dir <- dirname(parent.frame(2)$ofile) # source file dir
  setwd(this.dir)                            # set wd to source file dir
  code.path <- getwd()                       # define code path
  setwd('..')                                # set wd up one level
  setwd(paste(getwd(),'/output',sep=''))     # set wd to output folder
  output.path <- getwd()                     # define output path
  setwd('..')                                # set wd up one level
  setwd(paste(getwd(),'/input',sep=''))      # set wd to input folder
  input.path <- getwd()                      # define input path

  # Create output file:
  # date.time     <- gsub(' ','_',Sys.time())
  # date.time     <- gsub(':','-',date.time)
  # out.file.name <- paste(output.path,'/corona-bca_',date.time,'.out',sep='')
  out.file.name <- paste(output.path,'/corona-bca.out',sep='')
  outfile       <- file.create(out.file.name)

  # WRITE SCRIPT TO OUTPUT FILE:
  if(1==1){
    source.file.name <- paste(code.path,'/corona-bca.R',sep='')
    Rscript <- readLines(source.file.name)
    for(i in 1:length(Rscript)){cat('\n',Rscript[i],file=out.file.name,append=T)}
    cat('\n\n|-----------------------------------------------------------------------------|',file=out.file.name,append=T)
    cat('\n| R SCRIPT ABOVE |',file=out.file.name,append=T)
    cat('\n|-----------------------------------------------------------------------------|',file=out.file.name,append=T)
    cat('\n| R OUTPUT BELOW |',file=out.file.name,append=T)
    cat('\n|-----------------------------------------------------------------------------|\n',file=out.file.name,append=T)
  }
}

#------------------------------------------------------------------------------
# FUNCTIONS:
#------------------------------------------------------------------------------
{
  SIR.fn <- function(parms){

    # Unpack parameters from input list:
```

```r
    {
      T          <- parms$T
      dT         <- parms$dT
      N          <- parms$N
      R0         <- parms$R0
      gamma      <- parms$gamma
      rho.lo     <- parms$rho.lo
      rho.hi     <- parms$rho.hi
      threshold  <- parms$threshold
      I.0        <- parms$I.0
      VSL        <- parms$VSL
      sd         <- parms$sd
      GDP.now    <- parms$GDP.now
      g          <- parms$g
      shock.1    <- parms$shock.1
      shock.2    <- parms$shock.2
      r          <- parms$r
    }

    beta <- R0*gamma/(N*(1-rho.lo)) # Keeling and Rohini 2008

    XX <- matrix(dT,T/dT,1)
    S <- XX
    I <- XX
    R <- XX
    D <- XX
    c <- XX
    D2<- XX

    S[1] <- N
    I[1] <- I.0
    t <- 0
    for(tt in seq(dT,T,dT)){t <- t + 1
      S[t+1] <- S[t] - beta*I[t]*S[t] * dT

      # I[t+1] <- I[t] + beta*I[t]*S[t] * dT -
      #           (I[t]>threshold)*gamma/(1-rho.hi)*I[t] * dT -
      #           (I[t]<threshold)*gamma/(1-rho.lo)*I[t] * dT

      # I[t+1] <- I[t] + beta*I[t]*S[t] * dT -
      #   (I[t]>threshold)* ( gamma/(1-rho.hi)*(I[t]-threshold) +
      gamma/(1-rho.lo)*threshold ) * dT -
      #   (I[t]<threshold)*gamma/(1-rho.lo)*I[t] * dT

      I[t+1] <- I[t] + (beta*I[t]*S[t] - gamma*I[t]) * dT -
        (I[t]>=threshold)* ( gamma*rho.hi/(1-rho.hi)*(I[t]-threshold) + gamma*rho.lo/(1-
        rho.lo)*threshold ) * dT -
        (I[t]<threshold)*gamma*rho.lo/(1-rho.lo)*I[t] * dT

      R[t+1] <- R[t] + gamma*I[t] * dT
      D[t]   <- (I[t]>=threshold)*(rho.hi/(1-rho.hi)*gamma*(I[t]-threshold) +
                                  rho.lo/(1-rho.lo)*gamma*threshold) * dT +
                (I[t]< threshold)*rho.lo/(1-rho.lo)*gamma*I[t] * dT

      c[t]   <- beta*I[t]*S[t] * dT # new cases
    }
    S <- S[1:length(XX)]
    I <- I[1:length(XX)]
    R <- R[1:length(XX)]
    D <- D[1:length(XX)]
    c <- c[1:length(XX)]
    D2<- D2[1:length(XX)]

    return(list(S,I,R,D,c,D2))

  }
}

#-------------------------------------------------------------------------------
```

```
# MAIN PROGRAM:
#-----------------------------------------------------------------------------

# PARAMETERS:
{
  T         <- 365       # time horizon [days]
  dT        <- .1        # time step for numerical integration [days]
  N         <- 327*10^6  # U.S. population
  R0        <- 2.4       # Basic reproductive rate [2.4]
  gamma     <- 1/6.5     # recovery rate [1/6.5]
  rho.lo    <- .005      # Pr death if infected below threshold [.005]
  rho.hi    <- .015      # Pr death if infected above threshold [.015]
  I.0       <- 4125      # initial condition
  threshold <- 36*10^6   # health care system capacity, number of infection cases that can receive high standard of care
  VSL       <- 10*10^6   # Value of statistical life [US$]
  sd        <- .38       # fractional reduction in contact rate from social distancing
  GDP.now   <- 20*10^12  # current GDP [US$]
  g         <- .0175     # GDP growth rate [/yr]
  shock.1   <- .02       # fraction of GDP lost when outbreak is uncontrolled [.02]
  shock.2   <- .062      # fraction of GDP lost when outbreak is controlled [.062]
  eta       <- 1         # uncontrolled recovery rate convergence parameter
  r         <- .03       # discount rate [\yr]
  T.GDP     <- 30        #

  # Pack up parameters into a list:
  {
    parms              <- list(T)
    parms$T            <- T
    parms$dT           <- dT
    parms$N            <- N
    parms$R0           <- R0
    parms$gamma        <- gamma
    parms$rho.lo       <- rho.lo
    parms$rho.hi       <- rho.hi
    parms$I.0          <- I.0
    parms$threshold    <- threshold
    parms$VSL          <- VSL
    parms$sd           <- sd
    parms$GDP.now      <- GDP.now
    parms$g            <- g
    parms$shock.1      <- shock.1
    parms$shock.2      <- shock.2
    parms$eta          <- eta
    parms$r            <- r
    parms$T.GDP        <- T.GDP
    parms$output.path <- output.path
    parms0 <- parms
  }

}

# SCENARIO FUNCTION:
scenario.fn <- function(parms,figswitch,tabswitch){

  # Unpack parameters from input list:
  {
    T           <- parms$T
    dT          <- parms$dT
    N           <- parms$N
    R0          <- parms$R0
    gamma       <- parms$gamma
    rho.lo      <- parms$rho.lo
    rho.hi      <- parms$rho.hi
    threshold   <- parms$threshold
    I.0         <- parms$I.0
    VSL         <- parms$VSL
    sd          <- parms$sd
    GDP.now     <- parms$GDP.now
```

```
    g           <- parms$g
    shock.1     <- parms$shock.1
    shock.2     <- parms$shock.2
    eta         <- parms$eta
    r           <- parms$r
    T.GDP       <- parms$T.GDP
    output.path <- parms$output.path


    # Goldman Sachs projection hardwired:
    g.0       <- matrix(g,T.GDP,1)
    g.2       <- rbind(matrix(c(-shock.2,
                                 shock.2*.055/.062,
                                 shock.2*.035/.062,
                                 shock.2*.020/.062),4,1),
                       matrix(g,T.GDP-4,1))

    g.2       <- rbind(matrix(c(-.062,
                                 .055,
                                 .035,
                                 .020),4,1),
                       matrix(g,T.GDP-4,1))
  }

  # SIR model:
  {
    outs  <- SIR.fn(parms)
    S1    <- outs[[1]] # susceptible
    I1    <- outs[[2]] # infected
    R1    <- outs[[3]] # recovered
    D1    <- outs[[4]] # deaths
    c1    <- outs[[5]] # new infection cases

    # Reduced contact rate due to social distancing:
    parms$R0  <- R0*(1-sd)

    outs  <- SIR.fn(parms)
    S2    <- outs[[1]]
    I2    <- outs[[2]]
    R2    <- outs[[3]]
    D2    <- outs[[4]]
    c2    <- outs[[5]]
  }

  # GDP CURVES:
  {
    years  <- seq(0,T.GDP,1)
    GDP.0  <- matrix(0,length(years),1) # GDP path if no outbreak
    GDP.1  <- matrix(0,length(years),1) # GDP path if outbreak uncontrolled
    GDP.2  <- matrix(0,length(years),1) # GDP path if outbreak controlled

    # All GDP paths start at same point
    GDP.0[1] <- GDP.now
    GDP.1[1] <- GDP.now
    GDP.2[1] <- GDP.now

    # Growth without outbreak:
    for(t in 2:T.GDP){
      GDP.0[t] <- GDP.0[t-1]*(1+g.0[t])
    }

    # Growth with controlled outbreak based on GS forecast:
    for(t in 2:T.GDP){
      GDP.2[t] <- GDP.2[t-1]*(1+g.2[t-1])
    }

    # Growth with controlled outbreak adjusted for different assumed initial shock:
    for(t in 1:T.GDP){
      GDP.2[t] <- GDP.0[t] - (GDP.0[t]-GDP.2[t])*shock.2/(-g.2[1])
```

```
    }

    # Growth with uncontrolled outbreak:
    GDP.1[2] <- GDP.now * (1-shock.1)
    for(t in 3:T.GDP){
      GDP.1[t] <- GDP.0[t] - (GDP.0[t]-GDP.2[t])* ((GDP.0[2]-GDP.1[2])/(GDP.0[2]-GDP.2[2]))^eta
    }

    PV.0 <- 0
    PV.1 <- 0
    PV.2 <- 0
    for(t in 1:T.GDP){
      PV.0 <- PV.0 + GDP.0[t]/(1+r)^(t-1)
      PV.1 <- PV.1 + GDP.1[t]/(1+r)^(t-1)
      PV.2 <- PV.2 + GDP.2[t]/(1+r)^(t-1)
    }

  }

  # CREATE FIGURES:
  if(figswitch==1){

    # Infection curves:
    {
      tikz(paste(output.path,'/corona_fig_1A.tex',sep=''),
           width      = 3.2,
           height     = 3.2,
           pointsize  = 12,
           standAlone = TRUE)
      par(mar=c(4,4.5,1,1)) # figure border whitespace [bottom,left,top,right]

      bottom <- 0
      top    <- max(c(I1,I2))*1.1
      left   <- 0
      right  <- 300

      plot(seq(dT,T,dT),I2,type='l',col='black',
           main='',
           xlab='',
           ylab='',
           axes=FALSE,
           cex=1,
           xlim=c(left,right),
           ylim=c(bottom,top),
           cex.axis=.8,
           cex.lab =.5)

      lines(seq(dT,T,dT),I1,lty=2,col='black')
      lines(seq(dT,T,dT),threshold*matrix(1,T/dT,1),lty=3,col='black')

      axis(1,at=seq(0,350,50),las=1,labels=TRUE,cex.axis=.65) # Draw x axis
      axis(2,                 las=1,labels=TRUE,cex.axis=.65) # Draw y axis

      mtext('Number infected',side=2, line=3.25, cex=.8, las=0) # y-axis label
      mtext('$t$ [days]'     ,side=1, line=2.50, cex=.8, las=1) # x-axis label

      legend(left+(right-left)*.6,
             bottom+(top-bottom)*.9,
             legend=c('Controlled','Uncontrolled','Threshold'),
             col=c('black','black','black'),
             lty=c(1,2,3),
             cex=.6)

      text(left+(right-left)*.1,bottom+(top-bottom)*1,'(A)',cex=.8)

      dev.off()
    }

```

```
      # GDP curves:
      {
        tikz(paste(output.path,'/corona_fig_1B.tex',sep=''),
             width      = 3.2,
             height     = 3.2,
             pointsize  = 12,
             standAlone = TRUE)

        par(mar=c(4,4.5,1,1)) # figure border whitespace [bottom,left,top,right]

        top    <- GDP.now*1.1
        bottom <- GDP.now*0.9
        left   <- 0
        right  <- 5

        plot(seq(0,5,1),GDP.2[1:6],type='l',col='black',
             main='',
             xlab='',
             ylab='',
             axes=FALSE,
             cex=1,
             ylim = c(bottom,top),
             xlim = c(left,right),
             cex.axis=.8,
             cex.lab =.5)

        lines(seq(0,5,1),GDP.0[1:6],lty=3,col='black')
        lines(seq(0,5,1),GDP.1[1:6],lty=2,col='black')

        axis(1,at=seq(0,5,1),las=1,labels=TRUE,cex.axis=.65) # Draw x axis
        axis(2,              las=1,labels=TRUE,cex.axis=.65) # Draw y axis

        mtext('GDP [\\$]',side=2, line=3.5, cex=.8, las=0) # y-axis label
        mtext('$t$ [yrs]', side=1, line=2.5, cex=.8, las=1) # x-axis label

        legend(left+(right-left)*.6,
               bottom+(top-bottom)*.3,
               legend=c('No outbreak','Controlled','Uncontrolled'),
               col=c('black','black','black'),
               lty=c(3,1,2),
               cex=.6)

        text(left+(right-left)*.1,bottom+(top-bottom)*1,'(B)',cex=.8)

        dev.off()
      }

    }

    # PRINT TABLE OF RESULTS TO OUTPUT FILE:
    if(tabswitch==1){
      cat('\n================================================================\n',file=out.file.name,append=TRUE)
      cat('                         Uncontrolled   Controlled        Delta\n',file=out.file.name,append=TRUE)
      cat('--------------------------------------------------------------\n',file=out.file.name,append=TRUE)
      cat('Infections              ',
          sprintf('%12.4g %12.4g %12.4g\n',sum(c1),sum(c2),sum(c1)-sum(c2)),file=out.file.name,append=TRUE)
      cat('Deaths                  ',
          sprintf('%12.4g %12.4g %12.4g\n',sum(D1),sum(D2),sum(D1)-sum(D2)),file=out.file.name,append=TRUE)
      cat('PV of GDP loss          ',
          sprintf('%12.4g %12.4g %12.4g\n',PV.0-PV.1,PV.0-PV.2,PV.2-PV.1),file=out.file.name,append=TRUE)
      cat('Value of lives lost     ',
          sprintf('%12.4g %12.4g %12.4g\n',VSL*sum(D1),VSL*sum(D2),VSL*(sum(D1)-sum(D2))),file=out.file.name,append=TRUE)
```

```r
      cat('Total value lost        ',
          sprintf('%12.4g %12.4g %12.4g\n',PV.0-PV.1+VSL*sum(D1),PV.0-PV.2+VSL*sum(D2),
          PV.2-PV.1+VSL*(sum(D1)-sum(D2))),file=out.file.name,append=TRUE)
      cat('================================================================\n',file=
      out.file.name,append=TRUE)
    }

    return(list(I1,I2,c1,c2,D1,D2,PV.0,PV.1,PV.2,GDP.0,GDP.1,GDP.2))
  }

  # BENCHMARK RUN:
  outs <- scenario.fn(parms,1,1)

  # CREATE FIGURE 2 WITH 3 GDP CURVES:
  if(1==1){
    parms <- parms0
    parms$eta <- 1
    outs <- scenario.fn(parms,0,0)
    GDP.0      <- outs[[10]]
    GDP.2      <- outs[[12]]
    GDP.1.same <- outs[[11]]
    parms <- parms0
    parms$eta <- 2
    outs <- scenario.fn(parms,0,0)
    GDP.1.fast <- outs[[11]]
    parms <- parms0
    parms$eta <- .5
    outs <- scenario.fn(parms,0,0)
    GDP.1.slow <- outs[[11]]

    # Three GDP curves:
    {
      tikz(paste(output.path,'/corona_fig_2.tex',sep=''),
           width      = 3.2,
           height     = 3.2,
           pointsize  = 12,
           standAlone = TRUE)

      par(mar=c(4,4.5,1,1)) # figure border whitespace [bottom,left,top,right]

      top    <- GDP.now*1.1
      bottom <- GDP.now*0.9
      left   <- 0
      right  <- 5

      plot(seq(0,5,1),GDP.2[1:6],type='l',col='black',
           main='',
           xlab='',
           ylab='',
           axes=FALSE,
           cex=1,
           ylim = c(bottom,top),
           xlim = c(left,right),
           cex.axis=.8,
           cex.lab =.5)

      lines(seq(0,5,1),GDP.0[1:6],lty=3,col='black')
      lines(seq(0,5,1),GDP.1.fast[1:6],lty=2,col='gray')
      lines(seq(0,5,1),GDP.1.slow[1:6],lty=2,col='gray')
      lines(seq(0,5,1),GDP.1.same[1:6],lty=2,col='black')

      axis(1,at=seq(0,5,1),las=1,labels=TRUE,cex.axis=.65) # Draw x axis
      axis(2,              las=1,labels=TRUE,cex.axis=.65) # Draw y axis

      mtext('GDP [\\$]',side=2, line=3.5, cex=.8, las=0) # y-axis label
      mtext('$t$ [yrs]', side=1, line=2.5, cex=.8, las=1) # x-axis label

      legend(left+(right-left)*.4,
             bottom+(top-bottom)*.3,
```

```
           legend=c('No outbreak','Controlled','Uncontrolled benchmark','Uncontrolled fast','Uncontrolled slow'),
           col=c('black','black','black','gray','gray'),
           lty=c(3,1,2,2,2),
           cex=.6)

    dev.off()
  }

  eta <- parms0$eta
}

# FIND BREAK-EVEN VALUES:
if(1==1){
  # R0
  if(1==1){
    cat('Finding break-even value for R0\n')
    parms <- parms0
    R0.range <- seq(1.0,7,.01)
    NB  <- matrix(0,length(R0.range),1)
    TD1 <- matrix(0,length(R0.range),1)
    TD2 <- matrix(0,length(R0.range),1)
    for(R0 in R0.range){
      parms$R0 <- R0
      i    <- which(R0.range==R0)
      outs <- scenario.fn(parms,0,0)
      D1   <- outs[[5]]
      D2   <- outs[[6]]
      PV.1 <- outs[[8]]
      PV.2 <- outs[[9]]
      NB[i]  <- PV.2-PV.1+parms0$VSL*sum(D1)-parms0$VSL*sum(D2)
      TD1[i] <- sum(D1)
      TD2[i] <- sum(D2)
    }
    pdf(paste(output.path,'/fig_be_R0.pdf',sep=''))
    plot(R0.range,NB,type='l')
    lines(R0.range,matrix(0,length(R0.range),1),lty=2)
    dev.off()
    temp   <- which(NB>0)
    R0.be1 <- R0.range[min(temp)]
    R0.be2 <- R0.range[max(temp)]
    lo     <- min(temp);if(lo==1)          {R0.be1 <- -999}
    hi     <- max(temp);if(hi==length(NB)){R0.be2 <-  999}

    pdf(paste(output.path,'/fig_TD_R0.pdf',sep=''))
    plot(R0.range,TD1,type='l')
    lines(R0.range,TD2,lty=2)
    lines(R0.range,TD1-TD2,lty=3)
    dev.off()

  }

  # Save Figure 4:
  if(1==1){
    tikz(paste(output.path,'/corona_fig_4.tex',sep=''),
         width      = 3,
         height     = 3,
         pointsize  = 12,
         standAlone = TRUE)

    par(mar=c(3.5,3.5,1,1)) # figure border whitespace [bottom,left,top,right]

    left   <- min(R0.range)
    right  <- max(R0.range)
    top    <- max(TD1/10^6)
    bottom <- 0

    plot(R0.range,TD1/10^6,
         type='l',
```

```
          xlab='',
          ylab='',
          axes=FALSE,
          cex.axis=.8,
          cex.lab=.5)

      lines(R0.range,TD2/10^6,lty=2)
      lines(R0.range,(TD1-TD2)/10^6,lty=3)
      # lines(c(parms0$R0,parms0$R0),c(0,top),lty=2,col='gray')

      axis(1,las=1,labels=TRUE,cex.axis=.65) # Draw x axis
      axis(2,las=1,labels=TRUE,cex.axis=.65) # Draw y axis

      mtext('Total deaths [million]',side=2, line=2.2, cex=.65, las=0) # y-axis label
      mtext('R$_0$',                side=1, line=2.0, cex=.65, las=1) # x-axis label

      legend(left+(right-left)  *0.0,
             bottom+(top-bottom)*1.0,
             legend=c('uncontrolled','controlled','difference'),
             col=c('black','black','black'),
             lty=c(1,2,3),
             cex=.5)

      #text(left+(right-left)*.1,bottom+(top-bottom)*.9,'(A)',cex=.8)

      dev.off()
    }

    # rho.lo
    if(1==1){
      cat('Finding break-even value for rho.lo\n')
      parms <- parms0
      rho.lo.range <- seq(0,rho.hi,.0001)
      NB <- matrix(0,length(rho.lo.range),1)
      for(rho.lo in rho.lo.range){

        parms$rho.lo <- rho.lo
        outs <- scenario.fn(parms,0,0)
        D1   <- outs[[5]]
        D2   <- outs[[6]]
        PV.1 <- outs[[8]]
        PV.2 <- outs[[9]]
        NB[which(rho.lo.range==rho.lo)] <- PV.2-PV.1+parms0$VSL*sum(D1)-parms0$VSL*sum(D2)

      }
      pdf(paste(output.path,'/fig_be_rho.lo.pdf',sep=''))
      plot(rho.lo.range,NB,type='l')
      lines(rho.lo.range,matrix(0,length(rho.lo.range),1),lty=2)
      dev.off()
      temp   <- which(NB>0)
      rho.lo.be1 <- rho.lo.range[min(temp)]
      rho.lo.be2 <- rho.lo.range[max(temp)]
      lo     <- min(temp);if(lo==1)         {rho.lo.be1 <- -999}
      hi     <- max(temp);if(hi==length(NB)){rho.lo.be2 <-  999}
    }

    # rho.hi
    if(1==1){
      cat('Finding break-even value for rho.hi\n')
      parms <- parms0
      rho.hi.range <- seq(0,.05,.0001)
      NB <- matrix(0,length(rho.hi.range),1)
      for(rho.hi in rho.hi.range){

        parms$rho.hi <- rho.hi
        outs <- scenario.fn(parms,0,0)
        D1   <- outs[[5]]
        D2   <- outs[[6]]
        PV.1 <- outs[[8]]
```

```
        PV.2 <- outs[[9]]
        NB[which(rho.hi.range==rho.hi)] <- PV.2-PV.1+parms0$VSL*sum(D1)-parms0$VSL*sum(D2)

      }
      pdf(paste(output.path,'/fig_be_rho.hi.pdf',sep=''))
      plot(rho.hi.range,NB,type='l')
      lines(rho.hi.range,matrix(0,length(rho.hi.range),1),lty=2)
      dev.off()
      temp   <- which(NB>0)
      rho.hi.be1 <- rho.hi.range[min(temp)]
      rho.hi.be2 <- rho.hi.range[max(temp)]
      lo     <- min(temp);if(lo==1)          {rho.hi.be1 <- -999}
      hi     <- max(temp);if(hi==length(NB)){rho.hi.be2 <-  999}
    }

    # sd
    if(1==1){
      cat('Finding break-even value for sd\n')
      parms <- parms0
      sd.range <- seq(0,.9,.0005)
      NB <- matrix(0,length(sd.range),1)
      for(sd in sd.range){

        parms$sd <- sd
        outs <- scenario.fn(parms,0,0)
        D1   <- outs[[5]]
        D2   <- outs[[6]]
        PV.1 <- outs[[8]]
        PV.2 <- outs[[9]]
        NB[which(sd.range==sd)] <- PV.2-PV.1+parms0$VSL*sum(D1)-parms0$VSL*sum(D2)

      }
      pdf(paste(output.path,'/fig_be_sd.pdf',sep=''))
      plot(sd.range,NB,type='l')
      lines(sd.range,matrix(0,length(sd.range),1),lty=2)
      dev.off()
      temp   <- which(NB>0)
      sd.be1 <- sd.range[min(temp)]
      sd.be2 <- sd.range[max(temp)]
      lo     <- min(temp);if(lo==1)          {sd.be1 <- -999}
      hi     <- max(temp);if(hi==length(NB)){sd.be2 <-  999}
    }

    # VSL
    if(1==1){
      cat('Finding break-even value for VSL\n')
      parms <- parms0
      VSL.range <- seq(.5,20,.05)*10^6
      NB <- matrix(0,length(VSL.range),1)
      for(VSL in VSL.range){

        parms$VSL <- VSL
        outs <- scenario.fn(parms,0,0)
        D1   <- outs[[5]]
        D2   <- outs[[6]]
        PV.1 <- outs[[8]]
        PV.2 <- outs[[9]]
        NB[which(VSL.range==VSL)] <- PV.2-PV.1+VSL*sum(D1)-VSL*sum(D2)

      }
      pdf(paste(output.path,'/fig_be_VSL.pdf',sep=''))
      plot(VSL.range,NB,type='l')
      lines(VSL.range,matrix(0,length(VSL.range),1),lty=2)
      dev.off()
      temp    <- which(NB>0)
      VSL.be1 <- VSL.range[min(temp)]
      VSL.be2 <- VSL.range[max(temp)]
      lo      <- min(temp);if(lo==1)          {VSL.be1 <- -999}
      hi      <- max(temp);if(hi==length(NB)){VSL.be2 <-  999}
```

```r
    VSL <- parms0$VSL
  }

  # shock.1
  if(1==1){
    cat('Finding break-even value for shock.1\n')
    parms <- parms0
    shock.1.range <- seq(0,parms0$shock.2,.0005)
    NB <- matrix(0,length(shock.1.range),1)
    for(shock.1 in shock.1.range){

      parms$shock.1 <- shock.1
      outs <- scenario.fn(parms,0,0)
      D1   <- outs[[5]]
      D2   <- outs[[6]]
      PV.1 <- outs[[8]]
      PV.2 <- outs[[9]]
      NB[which(shock.1.range==shock.1)] <- PV.2-PV.1+parms0$VSL*sum(D1)-parms0$VSL*sum(D2)

    }
    pdf(paste(output.path,'/fig_be_shock1.pdf',sep=''))
    plot(shock.1.range,NB,type='l')
    lines(shock.1.range,matrix(0,length(shock.1.range),1),lty=2)
    dev.off()
    temp    <- which(NB>0)
    shock.1.be1 <- shock.1.range[min(temp)]
    shock.1.be2 <- shock.1.range[max(temp)]
    lo      <- min(temp);if(lo==1)          {shock.1.be1 <- -999}
    hi      <- max(temp);if(hi==length(NB)){shock.1.be2 <-  999}
  }

  # shock.2
  if(1==1){
    cat('Finding break-even value for shock.2\n')
    parms <- parms0
    shock.2.range <- seq(parms0$shock.1,.3,.0005)
    NB <- matrix(0,length(shock.2.range),1)
    for(shock.2 in shock.2.range){

      parms$shock.2 <- shock.2
      outs <- scenario.fn(parms,0,0)
      D1   <- outs[[5]]
      D2   <- outs[[6]]
      PV.1 <- outs[[8]]
      PV.2 <- outs[[9]]
      NB[which(shock.2.range==shock.2)] <- PV.2-PV.1+parms0$VSL*sum(D1)-parms0$VSL*sum(D2)

    }
    pdf(paste(output.path,'/fig_be_shock2.pdf',sep=''))
    plot(shock.2.range,NB,type='l')
    lines(shock.2.range,matrix(0,length(shock.2.range),1),lty=2)
    dev.off()
    temp    <- which(NB>0)
    shock.2.be1 <- shock.2.range[min(temp)]
    shock.2.be2 <- shock.2.range[max(temp)]
    lo      <- min(temp);if(lo==1)          {shock.2.be1 <- -999}
    hi      <- max(temp);if(hi==length(NB)){shock.2.be2 <-  999}
  }

  # threshold
  if(1==1){
    cat('Finding break-even value for threshold\n')
    parms <- parms0
    threshold.range <- seq(0,2*parms0$threshold,parms0$threshold/200)
    NB <- matrix(0,length(threshold.range),1)
    for(threshold in threshold.range){

```

```
        parms$threshold <- threshold
        outs <- scenario.fn(parms,0,0)
        D1   <- outs[[5]]
        D2   <- outs[[6]]
        PV.1 <- outs[[8]]
        PV.2 <- outs[[9]]
        NB[which(threshold.range==threshold)] <- PV.2-PV.1+parms0$VSL*sum(D1)-parms0$VSL*sum(D2)

      }
      pdf(paste(output.path,'/fig_be_threshold.pdf',sep=''))
      plot(threshold.range,NB,type='l')
      lines(threshold.range,matrix(0,length(threshold.range),1),lty=2)
      dev.off()
      temp    <- which(NB>0)
      threshold.be1 <- threshold.range[min(temp)]
      threshold.be2 <- threshold.range[max(temp)]
      lo      <- min(temp);if(lo==1)         {threshold.be1 <- -999}
      hi      <- max(temp);if(hi==length(NB)){threshold.be2 <-  999}
    }

    # r
    if(1==1){
      cat('Finding break-even value for r\n')
      parms <- parms0
      r.range <- seq(0,.1,.001)
      NB <- matrix(0,length(r.range),1)
      for(r in r.range){

        parms$r <- r
        outs <- scenario.fn(parms,0,0)
        D1   <- outs[[5]]
        D2   <- outs[[6]]
        PV.1 <- outs[[8]]
        PV.2 <- outs[[9]]
        NB[which(r.range==r)] <- PV.2-PV.1+parms0$VSL*sum(D1)-parms0$VSL*sum(D2)

      }
      pdf(paste(output.path,'/fig_be_r.pdf',sep=''))
      plot(r.range,NB,type='l')
      lines(r.range,matrix(0,length(r.range),1),lty=2)
      dev.off()
      temp  <- which(NB>0)
      r.be1 <- r.range[min(temp)]
      r.be2 <- r.range[max(temp)]
      lo    <- min(temp);if(lo==1)         {r.be1 <- -999}
      hi    <- max(temp);if(hi==length(NB)){r.be2 <-  999}
    }

    # T.GDP
    if(1==1){
      cat('Finding break-even value for T.GDP\n')
      parms <- parms0
      T.GDP.range <- seq(10,100,1)
      NB <- matrix(0,length(T.GDP.range),1)
      for(T.GDP in T.GDP.range){

        parms$T.GDP <- T.GDP
        outs <- scenario.fn(parms,0,0)
        D1   <- outs[[5]]
        D2   <- outs[[6]]
        PV.1 <- outs[[8]]
        PV.2 <- outs[[9]]
        NB[which(T.GDP.range==T.GDP)] <- PV.2-PV.1+parms0$VSL*sum(D1)-parms0$VSL*sum(D2)

      }
      pdf(paste(output.path,'/fig_be_T.pdf',sep=''))
      plot(T.GDP.range,NB,type='l')
      lines(T.GDP.range,matrix(0,length(T.GDP.range),1),lty=2)
```

```r
    dev.off()
    temp  <- which(NB>0)
    T.GDP.be1 <- T.GDP.range[min(temp)]
    T.GDP.be2 <- T.GDP.range[max(temp)]
    lo    <- min(temp);if(lo==1)         {T.GDP.be1 <- -999}
    hi    <- max(temp);if(hi==length(NB)){T.GDP.be2 <-  999}
  }
}

# PRINT TABLE OF BREAK-EVEN RESULTS TO OUTPUT FILE:
if(1==1){
  cat('\n==========================================================\n',file=out.file.name,append=TRUE)
  cat('                                Default         Break-even\n',file=out.file.name,append=TRUE)
  cat('----------------------------------------------------------\n',file=out.file.name,append=TRUE)
  cat('R0                        ',sprintf('%12.4g  (%6.3g,%6.3g)\n',parms0$R0,R0.be1,R0.be2),file=out.file.name,append=TRUE)
  cat('Low mortality rate        ',sprintf('%12.4g  (%6.3g,%6.3g)\n',parms0$rho.lo,rho.lo.be1,rho.lo.be2),file=out.file.name,append=TRUE)
  cat('High mortality rate       ',sprintf('%12.4g  (%6.3g,%6.3g)\n',parms0$rho.hi,rho.hi.be1,rho.hi.be2),file=out.file.name,append=TRUE)
  cat('Reduction in contact rate ',sprintf('%12.4g  (%6.3g,%6.3g)\n',parms0$sd,sd.be1,sd.be2),file=out.file.name,append=TRUE)
  cat('VSL                       ',sprintf('%12.4g  (%6.3g,%6.3g)\n',parms0$VSL,VSL.be1,VSL.be2),file=out.file.name,append=TRUE)
  cat('Uncontrolled shock        ',sprintf('%12.4g  (%6.3g,%6.3g)\n',parms0$shock.1,shock.1.be1,shock.1.be2),file=out.file.name,append=TRUE)
  cat('Controlled shock          ',sprintf('%12.4g  (%6.3g,%6.3g)\n',parms0$shock.2,shock.2.be1,shock.2.be2),file=out.file.name,append=TRUE)
  cat('Threshold                 ',sprintf('%12.4g  (%6.3g,%6.3g)\n',parms0$threshold,threshold.be1,threshold.be2),file=out.file.name,append=TRUE)
  cat('Discount rate             ',sprintf('%12.4g  (%6.3g,%6.3g)\n',parms0$r,r.be1,r.be2),file=out.file.name,append=TRUE)
  cat('Time horizon              ',sprintf('%12.4g  (%6.3g,%6.3g)\n',parms0$T.GDP,T.GDP.be1,T.GDP.be2),file=out.file.name,append=TRUE)
  cat('==========================================================\n',file=out.file.name,append=TRUE)
}

# NB=0 ISOQUANT shock.1 vs shock.2:
if(1==1){
  cat('Working on shock.1-shock.2 isoquant\n')
  nodes <- 30
  shock.1.0 <- shock.1; shock.1.range <- seq(0,.15,.15/(nodes-1))
  shock.2.0 <- shock.2; shock.2.range <- seq(0,.15,.15/(nodes-1))
  NBiso <- matrix(0,length(shock.2.range),length(shock.1.range))
  for(shock.1 in shock.1.range){
    for(shock.2 in shock.2.range){
      if(shock.2>=shock.1){
        parms <- parms0
        parms$shock.1 <- shock.1
        parms$shock.2 <- shock.2
        outs <- scenario.fn(parms,0,0)
        D1   <- outs[[5]]
        D2   <- outs[[6]]
        PV.1 <- outs[[8]]
        PV.2 <- outs[[9]]
        NBiso[which(shock.2.range==shock.2),which(shock.1.range==shock.1)] <-
          PV.2-PV.1+parms0$VSL*sum(D1)-parms0$VSL*sum(D2)
      }
    }
  }

  # Save figure:
  tikz(paste(output.path,'/corona_fig_iso_1.tex',sep=''),
       width      = 3,
       height     = 3,
```

```
       pointsize  = 12,
       standAlone = TRUE)

  par(mar=c(3.5,3.5,1,1)) # figure border whitespace [bottom,left,top,right]

  left   <- min(shock.2.range)
  right  <- max(shock.2.range)
  top    <- max(shock.1.range)
  bottom <- min(shock.1.range)

  contour(shock.2.range,shock.1.range,NBiso,levels=0,
          drawlabels=FALSE,
          xlab='',
          ylab='',
          axes=FALSE,
          cex.axis=.8,
          cex.lab=.5)

  points(parms0$shock.2,parms0$shock.1,pch=1,cex=.5)
  # lines(c(0,max(shock.1.range)),c(0,max(shock.1.range)),lty=2,col='gray')

  axis(1,las=1,labels=TRUE,cex.axis=.65) # Draw x axis
  axis(2,las=1,labels=TRUE,cex.axis=.65) # Draw y axis

  mtext('Uncontrolled shock',side=2, line=2.2, cex=.65, las=0) # y-axis label
  mtext('Controlled shock',  side=1, line=2.0, cex=.65, las=1) # x-axis label

  text(left+(right-left)*.1,bottom+(top-bottom)*.9,'(A)',cex=.8)

  dev.off()
}

# NB=0 ISOQUANT rho.hi vs threshold:
if(1==1){
  cat('Working on rho.hi-threshold isoquant\n')
  nodes <- 30
  rho.hi.0    <- rho.hi;     rho.hi.range    <- seq(0,.05,.05/(nodes-1))
  threshold.0 <- threshold; threshold.range <- seq(0,threshold.0*2,threshold.0*2/(nodes-
  1))
  NBiso <- matrix(0,length(threshold.range),length(rho.hi.range))
  for(rho.hi in rho.hi.range){
    for(threshold in threshold.range){
      parms <- parms0
      parms$rho.hi <- rho.hi
      parms$threshold <- threshold
      outs <- scenario.fn(parms,0,0)
      D1   <- outs[[5]]
      D2   <- outs[[6]]
      PV.1 <- outs[[8]]
      PV.2 <- outs[[9]]
      NBiso[which(threshold.range==threshold),which(rho.hi.range==rho.hi)] <-
        PV.2-PV.1+parms0$VSL*sum(D1)-parms0$VSL*sum(D2)
    }
  }

  # Save figure:
  tikz(paste(output.path,'/corona_fig_iso_2.tex',sep=''),
       width      = 3,
       height     = 3,
       pointsize  = 12,
       standAlone = TRUE)

  par(mar=c(3.5,3.5,1,1)) # figure border whitespace [bottom,left,top,right]

  left   <- min(threshold.range)
  right  <- max(threshold.range)
  top    <- max(rho.hi.range)
  bottom <- min(rho.hi.range)
```

```
  contour(threshold.range,rho.hi.range,NBiso,levels=0,
          drawlabels=FALSE,
          xlab='',
          ylab='',
          axes=FALSE,
          cex.axis=.8,
          cex.lab=.5)

  points(parms0$threshold,parms0$rho.hi,pch=1,cex=.5)

  axis(1,las=1,labels=TRUE,cex.axis=.65) # Draw x axis
  axis(2,las=1,labels=TRUE,cex.axis=.65) # Draw y axis

  mtext('Mortality rate above threshold',side=2, line=2.2, cex=.65, las=0) # y-axis label
  mtext('Heath care system threshold',  side=1, line=2.0, cex=.65, las=1) # x-axis label

  text(left+(right-left)*.1,bottom+(top-bottom)*.9,'(B)',cex=.8)

  dev.off()
}

# NB=0 ISOQUANT R0 vs sd:
if(1==1){
  cat('Working on R0-sd isoquant\n')
  nodes <- 50
  R0.0 <- R0; R0.range <- seq(1,7,(7-1)/(nodes-1))
  sd.0 <- sd; sd.range <- seq(0,1,(1-0)/(nodes-1))
  NBiso <- matrix(0,length(sd.range),length(R0.range))
  for(R0 in R0.range){
    for(sd in sd.range){
      parms <- parms0
      parms$R0 <- R0
      parms$sd <- sd
      outs <- scenario.fn(parms,0,0)
      D1   <- outs[[5]]
      D2   <- outs[[6]]
      PV.1 <- outs[[8]]
      PV.2 <- outs[[9]]
      NBiso[which(sd.range==sd),which(R0.range==R0)] <-
        PV.2-PV.1+parms0$VSL*sum(D1)-parms0$VSL*sum(D2)
    }
  }

  # Save figure:
  tikz(paste(output.path,'/corona_fig_iso_3.tex',sep=''),
       width      = 3,
       height     = 3,
       pointsize  = 12,
       standAlone = TRUE)

  par(mar=c(3.5,3,1,1)) # figure border whitespace [bottom,left,top,right]

  bottom <- min(R0.range)
  top    <- max(R0.range)
  right  <- max(sd.range)
  left   <- min(sd.range)

  contour(sd.range,R0.range,NBiso,levels=0,
          drawlabels=FALSE,
          xlab='',
          ylab='',
          axes=FALSE,
          cex.axis=.8,
          cex.lab=.5)

  points(parms0$sd,parms0$R0,pch=1,cex=.5)

  axis(1,las=1,labels=TRUE,cex.axis=.65) # Draw x axis
  axis(2,las=1,labels=TRUE,cex.axis=.65) # Draw y axis
```

```
  mtext('R$_0$',                           side=2, line=2.0, cex=.65, las=0) # y-axis label
  mtext('Social distancing effectiveness',side=1, line=2.0, cex=.65, las=1) # x-axis label

  text(left+(right-left)*.1,bottom+(top-bottom)*.9,'(C)',cex=.8)

  dev.off()
}

# NB=0 ISOQUANT R0 vs rho.hi:
if(1==1){
  cat('Working on R0-rho.hi isoquant\n')
  nodes <- 50
  NBiso <- matrix(0,length(rho.hi.range),length(R0.range))
  for(R0 in R0.range){
    for(rho.hi in rho.hi.range){
      parms <- parms0
      parms$R0 <- R0
      parms$rho.hi <- rho.hi
      outs <- scenario.fn(parms,0,0)
      D1   <- outs[[5]]
      D2   <- outs[[6]]
      PV.1 <- outs[[8]]
      PV.2 <- outs[[9]]
      NBiso[which(rho.hi.range==rho.hi),which(R0.range==R0)] <-
        PV.2-PV.1+parms0$VSL*sum(D1)-parms0$VSL*sum(D2)
    }
  }

  # Save figure:
  tikz(paste(output.path,'/corona_fig_iso_4.tex',sep=''),
       width      = 3,
       height     = 3,
       pointsize  = 12,
       standAlone = TRUE)

  par(mar=c(3.5,3,1,1)) # figure border whitespace [bottom,left,top,right]

  bottom <- min(R0.range)
  top    <- max(R0.range)
  right  <- max(rho.hi.range)
  left   <- min(rho.hi.range)

  contour(rho.hi.range,R0.range,NBiso,levels=0,
          drawlabels=FALSE,
          xlab='',
          ylab='',
          axes=FALSE,
          cex.axis=.8,
          cex.lab=.5)

  points(parms0$rho.hi,parms0$R0,pch=1,cex=.5)

  axis(1,las=1,labels=TRUE,cex.axis=.65) # Draw x axis
  axis(2,las=1,labels=TRUE,cex.axis=.65) # Draw y axis

  mtext('R$_0$',                           side=2, line=2.0, cex=.65, las=0) # y-axis label
  mtext('Mortality rate above threshold',side=1, line=2.0, cex=.65, las=1) # x-axis label

  text(left+(right-left)*.1,bottom+(top-bottom)*.9,'(D)',cex=.8)

  dev.off()
}
```
